# Community composition of ammonia-oxidizing archaea from surface and anoxic depths of oceanic oxygen minimum zones

**DOI:** 10.3389/fmicb.2013.00177

**Published:** 2013-07-01

**Authors:** Xuefeng Peng, Amal Jayakumar, Bess B. Ward

**Affiliations:** Department of Geosciences, Princeton UniversityPrinceton, NJ, USA

**Keywords:** ammonia-oxidizing archaea, oxygen minimum zone, Arabian sea, Eastern Tropical South Pacific, community composition, QPCR

## Abstract

Ammonia-oxidizing archaea (AOA) have been reported at high abundance in much of the global ocean, even in environments, such as pelagic oxygen minimum zones (OMZs), where conditions seem unlikely to support aerobic ammonium oxidation. Due to the lack of information on any potential alternative metabolism of AOA, the AOA community composition might be expected to differ between oxic and anoxic environments. This hypothesis was tested by evaluating AOA community composition using a functional gene microarray that targets the ammonia monooxygenase gene subunit A (*amoA*). The relationship between environmental parameters and the biogeography of the Arabian Sea and the Eastern Tropical South Pacific (ETSP) AOA assemblages was investigated using principal component analysis (PCA) and redundancy analysis (RDA). In both the Arabian Sea and the ETSP, AOA communities within the core of the OMZ were not significantly different from those inhabiting the oxygenated surface waters above the OMZ. The AOA communities in the Arabian Sea were significantly different from those in the ETSP. In both oceans, the abundance of archaeal *amoA* gene in the core of the OMZ was higher than that in the surface waters. Our results indicate that AOA communities are distinguished by their geographic origin. RDA suggested that temperature (higher in the Arabian Sea than in the ETSP) was the main factor that correlated with the differences between the AOA communities. Physicochemical properties that characterized the different environments of the OMZ and surface waters played a less important role, than did geography, in shaping the AOA community composition.

## Introduction

Nitrification plays a critical role in the marine nitrogen (N) cycle because it links the major sources (nitrogen fixation) and sinks (denitrification) of fixed reactive N by transforming ammonium to nitrite and subsequently nitrate. The importance of nitrification is also highlighted by its production of regenerated nitrate, which is taken up by phytoplankton, and which has implications for the estimate of export production from the euphotic zone of the ocean (Eppley and Peterson, 1979; Yool et al., 2007). Nitrification consists of two major steps, ammonia oxidation and nitrite oxidation, with ammonia oxidation considered to be the rate-limiting step (Kendall, 1998). Both steps are microbially mediated, although until recently, ammonia oxidation was thought to be accomplished only by ammonia-oxidizing bacteria (AOB). The discovery of the functional gene for ammonia oxidation, ammonia monooxygenase (*amo*), in archaea (Venter et al., 2004; Könneke et al., 2005; Treusch et al., 2005) led to the recognition that ammonia-oxidizing archaea (AOA) are ubiquitous in terrestrial and marine environments (Francis et al., 2005).

In several regions of the ocean, abundance of archaeal ammonia monooxygenase genes subunit A (*amoA*) was one to three orders of magnitude higher than the bacterial *amoA* gene abundance, enumerated by quantitative polymerase chain reaction (QPCR) (Wuchter et al., 2006a; Mincer et al., 2007; Agogue et al., 2008; Beman et al., 2008). Although the number of *amoA* gene copies per cell is reported to vary in the environment (Wuchter et al., 2006a; Agogue et al., 2008), the correlation between Thaumarchaeotal *amoA* and 16S rRNA gene abundances in the Arabian Sea indicates that most of the Thaumarchaeota are AOA (Pitcher et al., 2011). The sheer number of AOA relative to AOB suggests that they might be responsible for most of the ammonia-oxidation in the open ocean. The dominant role of AOA in ammonia-oxidation in the ocean is supported by a positive correlation between their abundance (implied from abundance of *amoA* or 16S rRNA genes) and ammonia-oxidation rates, observed in the Gulf of California (Beman et al., 2008), the North Sea (Wuchter et al., 2006a), and the coastal eastern Pacific (Santoro et al., 2010). Furthermore, there is evidence from the Southern California Bight (Ward, 1987), and the Gulf of California (Beman et al., 2008) that the abundance of AOB is decoupled from nitrification rates in the ocean.

However, a correlation between AOA abundance and ammonia oxidation rate is not always observed. In the Arabian Sea oxygen minimum zone (OMZ), Newell et al. (2011) found high abundances of AOA (>10^4^ copies ml^−1^) both within the oxygen deficient waters and below them (≥900 m depth), where ammonia oxidation rates were barely detectable. At an offshore Eastern Tropical South Pacific (ETSP) station, a local maximum of archaeal *amoA* gene abundance (>10^4^ copies ml^−1^) was found at 200 m in the OMZ where ammonia oxidation was not detected (Lam et al., 2009). In the Central California Current, AOA maintained high abundances at depths far below the bottom of the photic zone (on the order of 10^4^ *amoA* copies ml^−1^ down to 500 m), where ammonia oxidation rates were very low (10 nM day^−1^) (Santoro et al., 2010). This is intriguing because all known AOA (and AOB) are obligate aerobes. It is unknown what metabolism might support their growth in the Arabian Sea OMZ where neither oxygen nor ammonium is detectable, or in the deep water below the OMZ where ammonium supply and concentrations are very low, as are the measured ammonium oxidation rates.

Although there is no known alternative metabolism that might allow AOA to thrive in anoxic waters, AOA survival in OMZs could depend on unknown physiological capabilities. Such physiological differences might be reflected by differences in the composition of AOA communities in the anoxic depths of the OMZ relative to oxic waters. In order to assess whether the AOA community compositions within the OMZ differed from those above the OMZ, microarray analysis targeting the *amoA* gene was performed on DNA samples from both the Arabian Sea and the ETSP. Environmental parameters such as temperature and nutrient concentrations at these sites were also investigated using redundancy analysis (RDA) to determine their roles in shaping the AOA habitats. In addition, the abundance of archaeal *amoA* genes was quantified using QPCR.

Besides the environmental effects, the role of geographic separation on determining the community composition of AOA was also considered. A growing body of research has shown that microorganisms vary in community composition and abundance on different spatial scales, such as those summarized by Martiny et al. (2006). A phylogenetic study on AOA has shown that geography has a strong effect on their diversity (Pester et al., 2012). We hypothesized that the community composition of AOA from the Arabian Sea should differ from that from the ETSP, and tested the hypothesis with the microarray data.

## Materials and methods

### Site description

**Figure 1:**
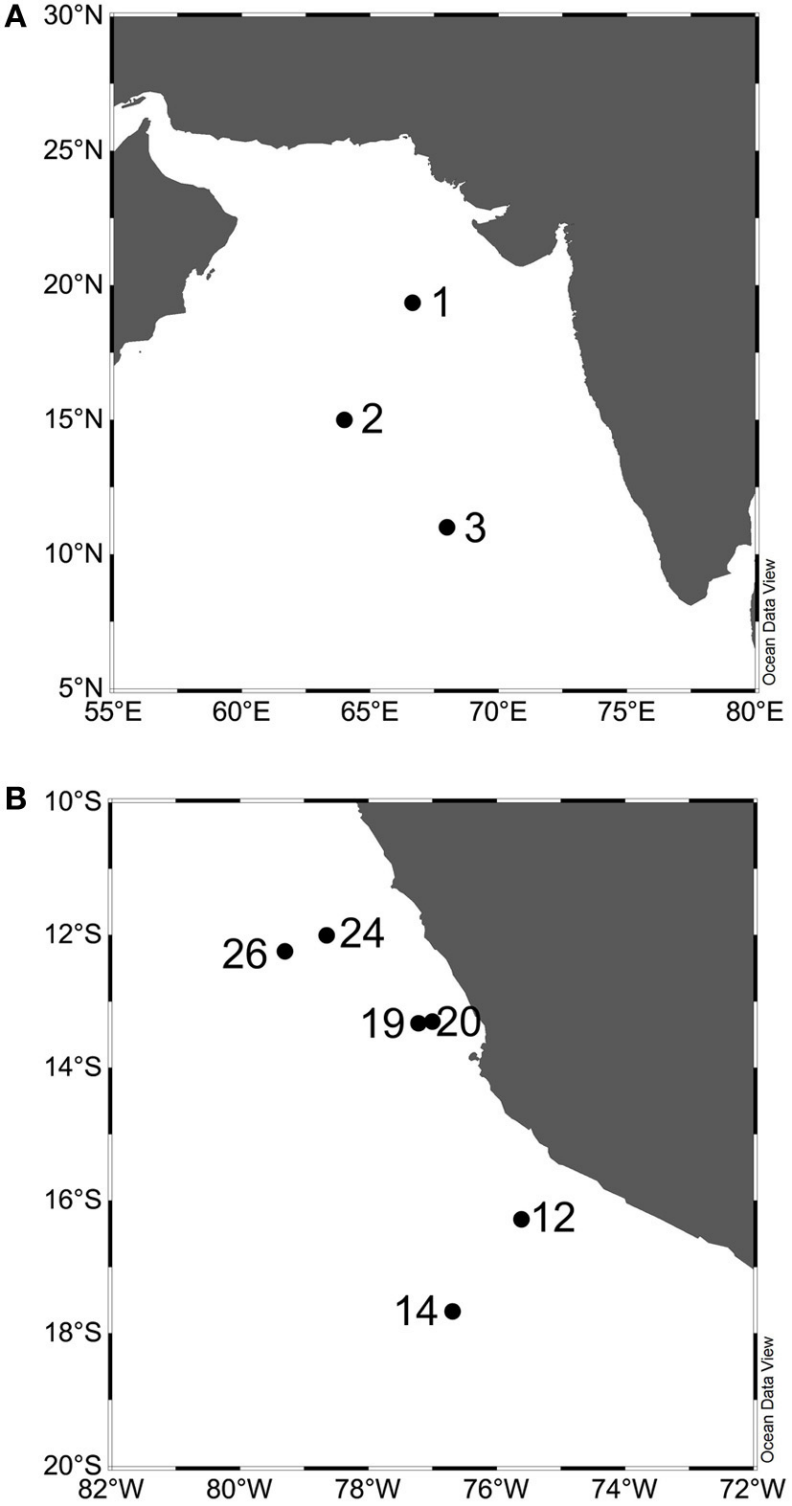
**Stations in the Arabian Sea (A) and the Eastern Tropical south Pacific (B)**. Maps were made using Ocean Data View (Schlitzer, 2012).

Samples from the Arabian Sea were collected as described by Newell et al. (2011) (Table 1). Briefly, in September 2007 on leg KNOX08 aboard the R/V Roger Revelle, samples were collected from above and within the anoxic depths at Stations 1, 2, and 3 in the open ocean OMZ. Stations 1 and 2 were within the permanent OMZ while Station 3 was on the periphery (Figure 1A; Newell et al., 2011). Samples from the ETSP were collected as described by Ward et al. (2009), in October 2005 aboard the R/V Knorr. Samples from above and within the anoxic core of the OMZ at six stations (12, 14, 19, 20, 24 and 26) off the coast of Peru were analyzed (Figure 1B, Table 1). Nutrient data for these sites has been reported previously (Ward et al., 2009; Bouskill et al., 2012).

**Table 1:** **Physicochemical data at the Arabian Sea and Eastern Tropical South Pacific stations**.

**Location**	**Station code**	**Latitude/Longitude**	**Sampling date**	**Depth (m)**	**Bottom depth (m)**	**Depth characteristics**	**Temp (°C)**	**Salinity (psu)**	**DO (μM)**	**NO^−^_2_ (μM)**	**NO^−^_3_ (μM)**	**Volume filtered (ml)**
**Arabian Sea**	Station 1	19° 22.98′ N, 66° 39.35′ E	September 2007	10	3151	Surface	28.77	36.65	203.0	0	0	9,400
				60		Chlorophyll max	23.20	36.10	22.2	0.2	11.4	10,400
				102		Oxycline	20.16	35.95	0.8	0.39	25.22	13,100
				150		Core of OMZ	17.87	35.76	1.1	5.24	15.92	11,610
				175		Core of OMZ	16.95	35.85	0.7	4.6	15.54	14,700
	Station 2	15° 00.00′ N, 64° 00.00′ E		10	3900	Surface	27.98	35.94	185.4	0	0.07	10,820
				150		Oxycline	20.14	35.95	0.6	0.57	22.36	12,800
				200		Core of OMZ	17.26	35.71	0.6	6.37	9.97	12,800
	Station 3	11° 00.00′ N, 68° 00.00′ E		10	4383	Surface	28.32	36.41	195.1	0	0.07	11,300
				110		Oxycline	20.79	35.85	1.2	0.06	25.76	12,600
				150		Core of OMZ	17.60	35.57	0.6	3.78	19.68	12,300
**Eastern Tropical**	Station 12	16° 16.86′ S, 75° 36.76′ W	October 2005	20	4263	Surface	*13.56*	*34.83*	*220.0*	*0.70*	*10*	4,300
**South Pacific**				260		Core of OMZ	10.80	34.74	2.0	0.54	22.20	8,600
	Station 14	17° 40.27′ S, 76° 41.27′ W		20	4200	Surface	13.56	34.83	*220.0*	0.00	2.04	4,300
				260		Core of OMZ	*10.80*	*34.74*	2.1	5.20	21.4	8,600
	Station 19	13° 19.99′ S, 77° 13.00′ W		20	1450	Surface	14.50	34.97	115.0	1.38	*19.60*	8,000
				260		Core of OMZ	11.16	34.81	2.0	2.55	*41.40*	8,600
	Station 20	13° 18.38′ S, 76° 59.87′ W		20	788	Surface	14.54	34.96	121.0	1.43	*12.50*	4,300
				260		Core of OMZ	11.49	34.83	2.1	4.38	*23.35*	4,000
	Station 24	12° 14.99′ S, 79° 18.00′ W		20	4930	Surface	16.96	34.96	235.0	0.80	*13.00*	4,300
				260		Core of OMZ	11.75	34.85	1.9	3.58	*26.9*	8,600
	Station 26	12° 00.50′ S, 78° 38.73′ W		20	3490	Surface	16.09	35.03	195.0	1.13	*11.70*	4,300
				260		Core of OMZ	11.20	35.03	2.1	5.21	*22.40*	8,600

*Values in italic fonts are interpolated from nearby depths profiles taken at the same stations. DO, dissolved oxygen*.

### Microarray hybridization and QPCR

Seawater samples (up to 13 L) were filtered onto 0.2 μm pore size Sterivex filters (Millipore, Billerica, MA) using a peristaltic pump, and filters were flash frozen in liquid nitrogen and stored at −80°C. Total DNA was extracted from Sterivex filters using either the Puregene DNA kit (Gentra, Minneapolis, MN) or the AllPrep DNA/RNA Mini Kit (Qiagen Sciences, Maryland, USA) with slight modifications (as in Ward, 2008) to the manufacturer's instructions. The extraction procedures were performed twice on each Sterivex filter in order to maximize the DNA yield.

The archetype array approach used in this study has been published previously (Bulow et al., 2008; Ward and Bouskill, 2011). Using an established algorithm (Bulow et al., 2008), 31 different archetypes were identified representing 1329 archaeal *amoA* sequences from GenBank at the time of probe design (November 2008). Each 90-mer oligonucleotide probe consisted of a 70-mer archetype sequence combined with a 20-mer reference oligo as an internal standard. Targets for microarray hybridization were prepared according to Ward and Bouskill (2011), hybridized in duplicate on a microarray slide and washed as described previously (Ward and Bouskill, 2011). Washed slides were scanned using a laser scanner 4200 (Agilent Technologies, Palo Alto, CA) and analyzed with GenePix Pro 6.0 (Molecular Devices, Sunnyvale, CA). All of the original array files are available at NCBI (National Center for Biotechnology Information) GEO (Gene Expression Omnibus; http://www.ncbi.nlm.nih.gov/geo/) database, accession GSE46851.

Archaeal *amoA* abundances were quantified using primers Arch-amoAF (5′-STAATGGTCTGGCTTAGACG-3′) and Arch-amoAR (5′-GCGGCCATCCATCTGTATGT-3′) (Francis et al., 2005). A plasmid containing an archaeal *amoA* fragment was constructed by TOPO TA Cloning (Invitrogen, Grand Island, NY). To make a standard curve, eight serial dilutions of the plasmid DNA were made and quantified using Quant-iT PicoGreen (Invitrogen, Grand Island, NY). Assays in triplicates were performed in a Stratagene Mx3000P QPCR system (Agilent Technologies, La Jolla, CA). Each 25-μ l reaction included 12.5 μ l of GoTaq qPCR Master Mix (Promega, Madison, WI), 0.4 μ M of each primer, 2 mM of MgCl_2_, and 2 ng of DNA template. The following thermal cycle was used to amplify archaeal *amoA* genes: 5 min of initial denaturation at 95°C, followed by 40 cycles of 94°C for 45 s, 53°C for 45 s, and 72°C for 1 min, and ending with 1 min at 95°C and a final elongation at 72°C for 15 min. All of the reactions were performed in a single 96-well plate. Dissociation curves of the QPCR products were checked to ensure the purity of the products. Cycle thresholds were determined automatically using MxPro QPCR Software (Agilent Technologies, La Jolla, CA). After calculating the number of archaeal *amoA* copies in each reaction, the final result was normalized to copies per milliliter of seawater (necessarily assuming 100% recovery) as:

$$\frac{\begin{array}{l} {}[\text{archaeal }amoA\text{ copy number }\times \text{ amount of DNA extracted}\\ \text{ from the Sterivex filter}(\text{ng})] \end{array}}{\begin{array}{l} \left[\text{amount of DNA used in the reaction }(\text{ng}\right)\\ \;\times \text{volume of seawater filtered }(\text{ml})] \end{array}}$$

### Data analysis

Quantification of hybridization signals was performed as described previously (Ward and Bouskill, 2011) with the following modifications. For each channel [532 nm (Cy3) and 635 nm (Cy5)], the average background fluorescence was recalculated after excluding background fluorescence values greater than the upper whisker of all the background fluorescence values. The upper whisker was defined as the 75th percentile plus 1.5 times the difference between the 25th and 75th percentiles. Such a filtering process was applied within each block on a microarray to account for variability in background fluorescence between blocks within an array. Another filter was applied to remove anomalous values of Cy3:Cy5 ratios among the triplicate features. This filter excluded any feature with a test statistic Z greater than 1.9 (CI = 80%) where Z is calculated as:

$$Z_{i}=\frac{r_{i}}{s/\sqrt{3}},\;i=1,\;2,\text{ or }3,$$

where r_*i*_ represents the ratio of Cy3 to Cy5, *s* the standard deviation of the three Cy3:Cy5 ratios. The raw microarray image was checked to ensure that the eliminated features actually exhibited anomalous hybridization signals. Then a normalized fluorescence ratio (FRn) for each archetype was calculated by dividing the fluorescence signal of the archetype by the highest fluorescence signal within the same array. The FRn of each archetype from the duplicate arrays were averaged. The relative fluorescence ratio (RFR) of each archetype was calculated as the contribution of FRn of the archetype to the sum of FRn of all AOA archetypes on the array. All of the data analyses above were performed using Microsoft Excel, and the following multivariate analyses on the array data were performed using R (R Core Team, 2012).

To explore the relationship between AOA communities from different stations and depths, a principal component analysis (PCA) was performed after the community composition data were transformed for chord distance (Legendre and Gallagher, 2001). Redundancy analysis (RDA) was performed using the FRn of each AOA archetype (after chord transformation) as the response variables, and temperature, dissolved oxygen (DO), nitrate and nitrite concentrations, and archaeal *amoA* abundance as explanatory variables. After a square root transformation, all explanatory variables were centered (divided by the standard deviation after the mean of each variable was subtracted). Depth and salinity were not included in the RDA because they had high linear dependence on temperature and dissolved oxygen. Linear dependencies between the environmental variables were examined by calculating variance inflation factors (VIF), and including one or both of these two variables in the RDA largely inflated VIFs of temperature and dissolved oxygen (>16) (Borcard et al., 2011). The VIFs of the five explanatory variables chosen for the RDA were reasonably low (from 2.4 to 4.2). Excluding depth and salinity from the RDA sacrificed a minimal amount of the variance captured by the first two axis of the RDA (<2%).

The hypothesis that the AOA community composition differed between surface depths and OMZ depths, in the Arabian Sea and in the ETSP respectively, was tested using Multi-response Permutation Procedure (MRPP) for its relaxed requirements on the data distribution and the convenience to relate the analysis visually to the biplots from PCA (Zimmerman et al., 1985). MRPP was also used to test the null hypothesis that the AOA community composition in the Arabian Sea was the same as that in the ETSP. A significance level of 5% was chosen.

## Results

### Physicochemical properties

There was a large temperature gradient (~10°C) between the surface and the core of the OMZ in the Arabian Sea, while the temperature difference was smaller in the ETSP (Table 1). The temperatures at sampled depths in the Arabian Sea were significantly higher than those in the ETSP (*P* < 0.001). The variability in salinity between different depths was small in both the Arabian Sea and the ETSP, but the salinities in the Arabian Sea were significantly higher than those in the ETSP (*P* < 0.001). Dissolved oxygen concentrations were below detection in the core of the OMZ where an accumulation of nitrite (up to 6.4 μ M) was observed in both the Arabian Sea and the ETSP.

### Archaeal AMOA gene abundance

**Figure 2:**
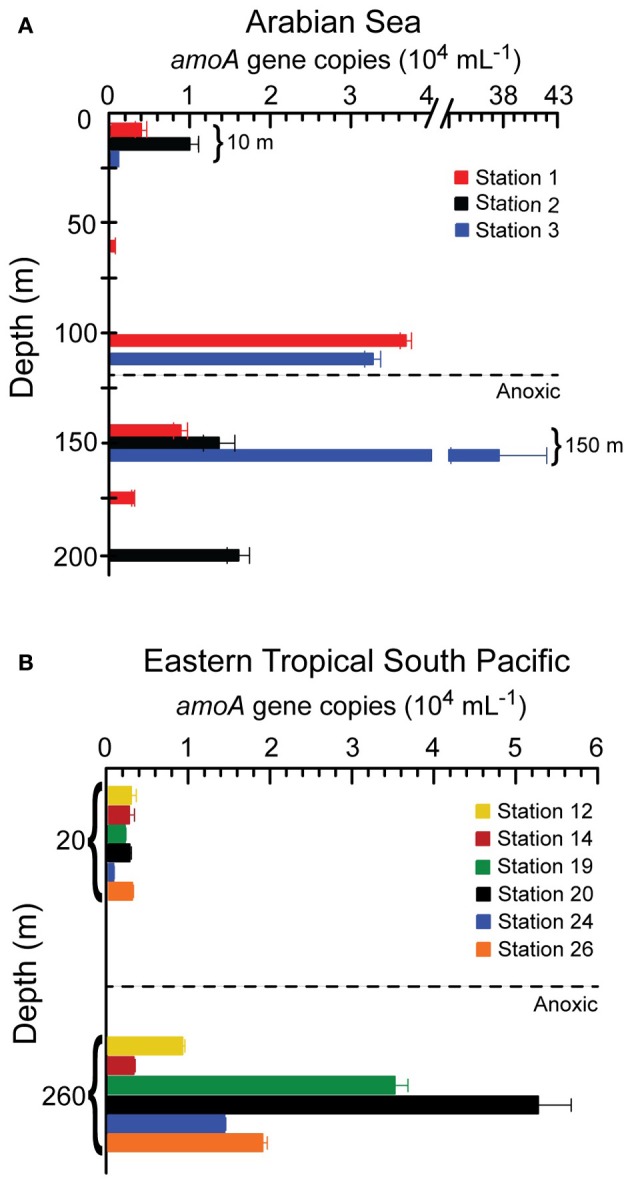
**Archaeal *amoA* abundance in copies mL^−1^ at different depths in the Arabian Sea (A) and the Eastern Tropical South Pacific (B)**. Error bars represent standard deviations of the triplicates in the QPCR run. In some cases the error bars were shorter than the width of the symbol and hence not visible. Different depths were sampled at different stations, so missing bars do not imply zero abundance, rather that samples were not analyzed at those depths.

Archaeal *amoA* gene abundances were lower in the surface (from just over 500 to just under 10,000 copies mL^−1^) than in the anoxic core of the OMZ (over 10,000 copies mL^−1^ in most samples) at all stations except at Station 1 in the Arabian Sea (Figure 2). At Station 1 in the Arabian Sea, archaeal *amoA* gene abundance was the lowest in the chlorophyll maximum (479 copies mL^−1^), and the highest at the bottom of the oxycline right above the anoxic depths (36,537 copies mL^−1^, Figure 2A). At Stations 2 and 3 in the Arabian Sea, the archaeal *amoA* gene abundance at the oxycline was much higher than that in the surface, reaching >10,000 copies mL^−1^. At both surface and the anoxic depths of the OMZ, the archaeal *amoA* gene abundances were comparable between the Arabian Sea and the ETSP (Figure 2).

### Community composition analysis

**Figure 3:**
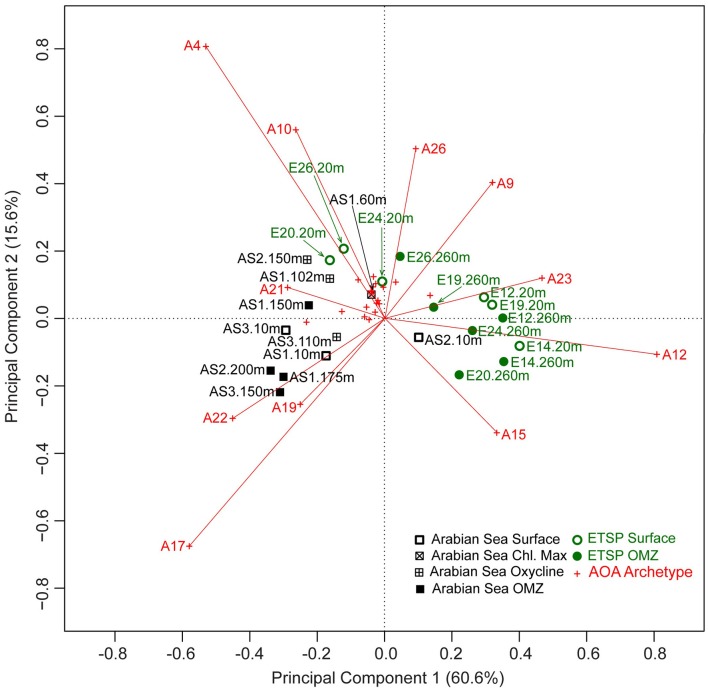
**Distance biplot of principal component analysis (PCA) on AOA community composition from the Arabian Sea and the ETSP using chord distance**. Each AOA archetype is shown as a red cross. AOA archetypes that had a relatively high contribution to the two principal components plotted were highlighted by labeling them with “A archetype number,” and drawing the vector between the origin and the AOA archetype. Circles represent samples from the ETSP (E), and squares represent samples from the Arabian Sea (AS). Hollow symbols represent samples from oxygenated surface waters; filled symbols represent samples from anoxic waters. Squares filled with a vertical cross represent samples from the oxycline, and the square filled with a rotated cross represents the sample from the chlorophyll maximum at Station 1 in the Arabian Sea. Distances among AOA communities are approximations of their Euclidean distance in the multidimensional space (Borcard et al., 2011).

In the Arabian Sea, three OMZ AOA assemblages clustered closely (AS1.175 m, AS2.200 m, and AS3.150 m). Nevertheless, the four communities from the anoxic core of the OMZ taken together were not significantly different from the surface communities (Table 2) because one OMZ community (AS1.150 m) was very dissimilar from the rest of the OMZ communities (Figure 3). AOA assemblages from the oxycline differed significantly from the OMZ assemblages in the Arabian Sea (*P* < 0.05, Table 2), even though the measured physicochemical properties in the oxycline were more similar to those in the OMZ than to those in the surface (Table 1). The AOA community from the chlorophyll maximum (AS1.60 m) was separated from most of the other AOA communities in the Arabian Sea, and it was ordinated closely to some of the AOA communities in the ETSP such as E24.20 m (Figure 3). One surface assemblage in the Arabian Sea (AS2.10 m) was the most similar to ETSP AOA communities (Figure 3).

**Table 2:** **Summary of *p* values of Multi-Response Permutation Procedure comparing different AOA communities pairwise**.

	**AS surface**	**AS Oxycline**	**AS OMZ**	**ETSP surface**
AS Oxycline	n.s.			
AS OMZ	n.s.	0.045		
ETSP surface	n.s.	n.s.	0.005	
ETSP OMZ	0.022	0.010	0.003	n.s.

*The p value is the probability of a smaller or equal test statistics (Zimmerman et al., 1985). Only the p values smaller than 0.05 were shown. AS: Arabian Sea. ETSP: Eastern Tropical South Pacific. OMZ: oxygen minimum zone, n.s.: not significant*.

In the ETSP, there was no significant separation between the AOA communities from the core of the OMZ and those from the surface (Figure 3, Table 2). Most AOA assemblages in the ETSP were characterized by positive values along the first principal component (PC1) (Figure 3). The AOA communities in the ETSP OMZ were significantly different from those in the Arabian Sea OMZ, oxycline, and surface, respectively (Table 2). Overall, AOA communities in the Arabian Sea were significantly different from those in the ETSP (*P* < 0.001), as evident in the PCA biplot (Figure 3). The first two components of PCA, which do not include any environmental variables, captured 76.2% of the variation of the AOA community structure (Figure 3).

**Figure 4:**
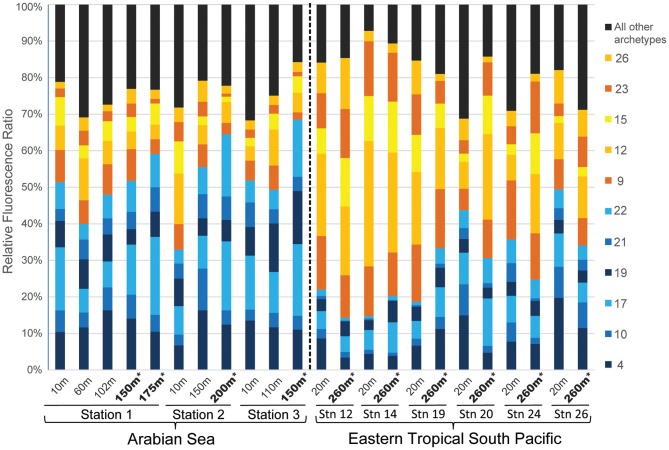
**Relative fluorescence ratios (RFR) of archaeal *amoA* archetypes from samples collected in the Arabian Sea and the ETSP**. Numbers in the legend represent the AOA archetype identification number. The AOA archetypes that were important constituents of the Arabian Sea communities were shown in blue, while the AOA archetypes that were important constituents of the ETSP communities were shown in orange. The rest of the AOA archetypes were grouped and shown in black. Samples from the anoxic depths were highlighted in bold and with an asterisk.

About a third (11 out of 30) of the AOA archetypes contributed strongly (labeled vectors) to the first two principal components (Figure 3). In fact, these 11 AOA archetypes contributed over 70% of the total community RFR in most of the samples (Figure 4). Six of them (AOA -4, -10, -17, -19, -21, and -22) contributed to a relatively larger percentage of the total community RFR in the Arabian Sea than in the ETSP (Figures 3 and 4). The other five of the 11 important archetypes (AOA -9, -12, -15, -23, and -26) were relatively more abundant in the ETSP than in the Arabian Sea.

**Figure 5:**
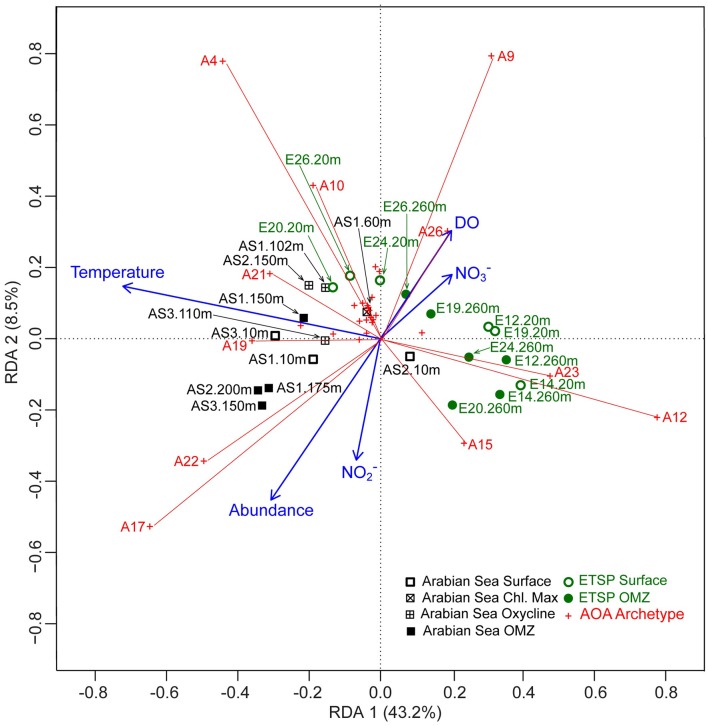
**Distance triplot of redundancy analysis (RDA) on AOA community composition from the Arabian Sea and the ETSP, using temperature, dissolved oxygen (DO), archaeal *amoA* abundance, nitrite concentration (NO^−^_2_), and nitrate concentration (NO^−^_3_) as explanatory variables**. Symbols used here for AOA archetypes and sample stations are the same as described in Figure 3. The blue arrows are the vectors of the explanatory variables. Distances among AOA communities are approximation of their Euclidean distance in the multidimensional space. The length of the projection of any sample onto an archetype approximates the RFR of the archetype in that sample. The angles between an environmental variable and an archetype reflect their correlations (Borcard et al., 2011).

The redundancy analysis (RDA) showed that temperature played an important role in the dispersion of the sites along the first axis (RDA1), which captured 43.2% of the variation of the dataset (Figure 5). Most AOA communities in the Arabian Sea were associated with higher temperature compared to AOA communities in the ETSP. The relative abundances of the group of AOA archetypes that were more abundant in the ETSP than in the Arabian Sea (AOA -9, -12, -15, -23, and -26) were positively correlated with dissolved oxygen and nitrate concentration. The group of AOA archetypes with higher RFR in the Arabian Sea than in the ETSP (AOA -4, -10, -17, -19, -21, and -22) were characterized by high temperature. When the number of archaeal *amoA* gene copies was high, archetypes AOA -17 and -22 made up a greater proportion of the AOA community than when the number of archaeal *amoA* gene copies was low. The opposite is true for archetypes AOA -9 and -26 (Figure 5).

## Discussion

Microarrays offer high throughput compared to other molecular methods such as building clone libraries (Ward and Bouskill, 2011) and therefore facilitate greater sample coverage and replication (Bouskill et al., 2012). In the archetype approach used here, each probe represents all sequences within 87 ± 3% of its 70-mer sequence (Taroncher-Oldenburg et al., 2003). Our knowledge of the ranges of environments that each AOA archetype represents has expanded since the development of the microarray used in this study due to rapid growth of the sequence database (Biller et al., 2012; Pester et al., 2012). The thousands of new AOA *amoA* sequences, which have been reported since the time of the array design, make it clear that the current array cannot represent the entire AOA diversity. Although most of the additional diversity has been reported from soils, there are many archetypes detected in marine samples that are not represented on the array. Therefore, the patterns we observed, based on the limited sequence database available in GenBank in 2009, can still provide valuable insight on AOA community composition and its variation in space and time, but cannot be extrapolated to unrepresented members of the AOA assemblage.

### Similarity in AOA community composition between the OXIC and the anoxic depths of the OMZ

Contrary to our hypothesis, AOA communities from oxic and anoxic depths of OMZs were not significantly different. The oxygen level at the OMZ core depth (260 m) in the ETSP has recently shown to be truly anoxic by highly sensitive STOX oxygen sensors with a detection limit of 10 nM (Thamdrup et al., 2012). Because molecular oxygen is required for aerobic ammonia oxidation, it was unlikely that the AOA communities found in anoxic depths were oxidizing ammonia aerobically. They might be simply inactive at a low energy state, or they might be capable of other metabolisms, which allow them to exist under oxygen-limiting conditions. For example, *Candidatus Nitrosoarchaeum limnia*, an AOA enriched from low salinity environments had a minimum oxygen requirement for growth between 29 and 59 μM (Mosier et al., 2012). The half-saturation concentration (*K*_m_) for oxygen of the only pure marine AOA isolate, *Nitrosopumilus maritimus*, was 3.9 μM (Martens-Habbena et al., 2009). A marine sedimentary AOA in coculture with sulfur-oxidizing bacteria was reported to have a similar *K*_m_, 2.0 μM (Park et al., 2010). Dalsgaard et al. (2013) reported an extremely low *K*_m_ (330 nM) for oxygen in a water sample from the anoxic depth in the ETSP. Despite their small genome and apparently restricted metabolic repertoire, AOA might be capable of multiple lifestyles across ecologically significant environmental variation. A recent study on the global distribution of AOA using over 6200 archaeal *amoA* gene sequences found no difference between the AOA in oxic and oxygen-limiting environments (Cao et al., 2013).

On the other hand, it is worth noting that differences in microbial diversity have been attributed to selective pressure from physical/chemical conditions at different depths of the water column in the OMZ by other authors. For example, a significant difference between the bacterial community structure of the surface and the anoxic core of the ETSP OMZ was revealed using 16S rRNA clone libraries at the class level (Stevens and Ulloa, 2008). The microarray data suggest that these same selective pressures are not sufficient to differentiate among AOA at the archetype level, which is defined by the most variable 70 bp region of all archaeal *amoA* sequences at the time of the array development. A phylogenetic study on the archaeal *amoA* sequences from the ETSP OMZ showed that although some AOA operational taxonomic units (OTUs) were present in both the well-oxygenated depths and the anoxic depths, a distinct cluster of AOA OTUs were found only in the anoxic depths of the permanent and seasonal OMZ (Molina et al., 2010).

Another possible explanation for the lack of difference between surface and deeper AOA communities is that there was some type of vertical exchange of AOA. Mixing is unlikely due to the strong stratification of the water column in the Arabian Sea and the ETSP (Rao et al., 1989; Fiedler and Talley, 2006). Autonomous movement by AOA is improbable, since most AOA strains reported so far do not exhibit motility (Könneke et al., 2005; Santoro and Casciotti, 2011; Tourna et al., 2011). The only strain reported to have the potential for motility was from low-salinity marine sediments (Mosier et al., 2012). In any case, individual cells simply cannot autonomously travel a distance on the scale of tens or hundreds of meters. There might be particle flux that could bring surface communities down into the OMZ. In the Eastern Tropical North Pacific, another major oceanic OMZ, downward flux of particle-associated bacterial nitrifiers was reported (Karl et al., 1984). It is possible that AOA investigated in this study were also associated with downward particle flux in the OMZs. If this was true, then the samples from the anoxic depths should have included AOA communities from the surface, which would lessen any potential difference in community composition between the oxic and the anoxic depths. In the Arabian Sea glycerol dialkyl glycerol tetraether (GDGT), a lipid produced by marine Crenarchaea, was associated with downward particle flux (Wuchter et al., 2006b). On the other hand, enumeration of AOA with catalyzed reporter deposition fluorescence *in situ* hybridization (CARD-FISH) showed that most of the marine Crenarchaea were free-living (Woebken et al., 2007). It seems likely that preferential association with particles would not be adaptive for the lifestyle of tiny autotrophic microbes, which is selective for the planktonic state. Thus the importance of vertical transport remains questionable.

### Archaeal AMOA abundance

Although no difference in AOA community structure was found between the surface and the anoxic core of the OMZ, there was a large difference in the abundance of archaeal *amoA* genes between these depths at most stations (Figure 2). The low abundance of archaeal *amoA* genes at surface depths was expected because AOA are inhibited by light (Merbt et al., 2012) and ammonia oxidation rates in the upper euphotic zone are usually very low. It is likely that our sampling missed the highest abundances of AOA because we did not sample the depth of the primary nitrite maximum, where local abundance maxima in numbers and rates are often reported (e.g., Beman et al., 2008; Coolen et al., 2007; Newell et al., 2011).

The high abundance of archaeal *amoA* gene in the anoxic core of the OMZs was enigmatic, but consistent with numerous previous reports in OMZs (e.g. Beman et al., 2008; Newell et al., 2011; Pitcher et al., 2011; Bouskill et al., 2012). On the other hand, rates of ammonia oxidation (Newell et al., 2011) at the same stations (different depths) showed the characteristic distribution of highest rates in the oxic layer and low to negligible rates within and below the OMZ. The rate maximum was generally deeper than the depth from which the array sample was collected so no direct comparisons can be made between rates and community composition. Still it is striking that the arrays detected essentially the same community composition across depths that likely varied a great deal in ammonia oxidation rates. The archaeal *amoA* gene abundances in the ETSP measured in this study were consistent with previously reported values in the same region. For example, Station 26 in the ETSP in this study was close to Station 7 from Lam et al. (2009), and the archaeal *amoA* gene abundances in the surface and the core of the OMZ reported in this study were very similar to those from Lam et al. (2009). The archaeal *amoA* gene abundances in the Arabian Sea measured in this study, although similar to those in the ETSP, were generally an order of magnitude lower compared to previous studies (Newell et al., 2011; Pitcher et al., 2011; Bouskill et al., 2012).

### Biogeography

AOA communities differed significantly between the Arabian Sea and the ETSP, suggesting that geographical variation exerts a strong control over the community structure of AOA. Among the four physicochemical variables investigated, temperature was the most important factor that distinguished the AOA communities in the Arabian Sea from those in the ETSP (Figure 5). This regional pattern is consistent with the findings of Pester et al. (2012) who analyzed AOA *amoA* genes in soil from Namibia, Costa Rica, Austria, and Greenland. They found that AOA community composition was different among these four locations, and geographic location on the continental scale had a strong effect on the presence or absence of different AOA taxa in individual soils. Pester et al. (2012) identified total nitrogen concentration, organic carbon content, and pH as major driving forces for AOA community structure in soils. However, in a biogeographic study on AOB in soils, temperature was most strongly correlated with AOB community structure among the suite of environmental variables measured (Fierer et al., 2009). Both this study and the study by Pester et al. (2012) lend support to the hypothesis that marine microplankton display biogeographic patterns. In a study that surveyed bacterioplankton communities using clone libraries in nine geographically distinct regions of the world ocean, 69% of the operational taxonomic units were endemic (Pommier et al., 2007).

Biller et al. (2012) investigated the factors correlated with global genotype distribution of AOA *amoA* based on over 8000 *amoA* sequences from literature and public databases. They found that, on the first level, habitat type accounted for the greatest variability in the dataset, separating AOA into 13 groups. On a second level, temperature, latitude, water depth, and salinity were significantly associated with AOA community composition, although the correlation was weak to moderate in the case of temperature and latitude. Their conclusions are consistent with the biogeographic separation between the AOA communities from the two different oceans in our study. All of our samples fell into the “ocean water column” habitat type on the first level, as defined by Biller et al. (2012). On the second level, temperature had the most pronounced influence on the distinction between the AOA communities analyzed in our study. Different types of AOA might each have a temperature optimum for growth, and this could lead to difference in community composition if the temperature optima for different AOA have little overlap. Due to the lack of direct investigation on the effect of temperature on the AOA community composition, it remains unclear how temperature determines the distribution of AOA. Since both sites in this study were in low latitudes (<23.4°, Table 1), the geographic distinction in the AOA communities found between the Arabian Sea and the ETSP indicates that variables other than latitude are important in determining AOA community composition.

### A closer look at community composition patterns at the archetype level

We found it intriguing that in the Arabian Sea, the AOA communities in the oxycline differed significantly from those in the OMZ, and that AOA communities in the OMZ were more closely related to surface AOA communities (Figure 3), even though the oxycline is characterized by physicochemical properties much more similar to the OMZ compared to the surface (Table 1). The community composition difference is largely attributable to high RFRs of AOA-4 and -10 but low RFRs of AOA-17, -19, and -22 in the oxycline AOA communities (Figure 3). Highest ammonium oxidation rates were reported previously from the oxycline region (Newell et al., 2011), suggesting that archetypes AOA-4 and AOA-10 might represent the most active groups.

The same collection of AOA archetype probes and microarray approach were used to assess AOA diversity in a wide range of marine environments including the Chesapeake Bay, Sargasso Sea, the North Atlantic, as well as the Arabian Sea and the ETSP (Bouskill et al., 2012). Geographic location was also the major factor that distinguished different AOA assemblages in their study. The AOA communities in the Arabian Sea were different from those in the ETSP in their study, but the AOA archetypes that characterized these two geographic locations (AOA-3, -11, -16, -20, -22, -25, -29, and -31 for the Arabian Sea; AOA-1, -2, -8, -10, -21, -23, -28, -30 for the ETSP) were mostly different from the important archetypes in our study. This might be explained by the fact that environmental variables were included in the unconstrained ordination (PCA) in Bouskill et al. (2012), which is not the case in our study where environmental variables were only included in the constrained ordination (RDA) (Legendre and Gallagher, 2001). Nevertheless, the AOA archetypes with the highest RFRs (AOA-9, -12, -4, -26, and -17) found by Bouskill et al. (2012) were also important in defining the community structure of AOA assemblages in the present study (Figures 3 and 4).

It is worth noting that archetype AOA-1, which represents the largest number of AOA sequences in GenBank at the time of the microarray development, contributed only minimally to all of the AOA communities in our study. Archetype AOA-1 represents the cultivated marine strain *N. maritimus* and a large number of sequences retrieved with primers designed using *N. maritimus* sequence (e.g., Francis et al., 2005; Beman and Francis, 2006; Santoro et al., 2008; Zhang et al., 2008). The underrepresentation of AOA-1 in our samples suggests that *N. maritimus* is not necessarily representative of the AOA assemblages in the global ocean. This is consistent with the finding that AOA clone libraries constructed with seawater samples from the Gulf of California did not recover any *N. maritimus*-like sequences (Beman et al., 2008). Therefore it is critical to isolate other AOA strains that are typical of marine environments, in order to better understand their physiology and factors that determine their ecology.

The PCR primers used here for quantification of AOA *amoA* were the same ones used by many investigators to build clone libraries, which are often dominated by *N. maritimus*-like sequences. The array targets, however, were prepared from whole DNA, i.e., without PCR amplification. Thus the two assays are apparently detecting different subsets of the overall assemblage.

### Beyond abiotic conditions

The first two axes of RDA together captured just over half (51.7%) of the variation of AOA community composition. This indicated that factors not included in our ordination should be important in controlling the AOA diversity. Strom (2008) pointed out the inadequacy of explanations of microbial diversity based on “resource availability and abiotic conditions,” and she proposed that community interactions such as mortality, allelopathy, and symbiosis warranted more consideration because they have “strong selective pressure on marine microbes.” In our study, archaeal *amoA* gene abundance could be regarded as an index for community interaction among AOA. In other words, the interaction among AOA in a community with high AOA abundance should presumably be different than that in a community with low AOA abundance. From our model, we can see that archetypes AOA -17 and -22 were positively correlated with AOA abundance (Figure 5). In three samples from the anoxic core of the Arabian Sea with high archaeal *amoA* abundance (175 m at Station 1, 200 m at Station 2, and 150 m at Station 3), archetypes AOA -17 and -22 made up over a third of the total AOA community (Figure 4), suggesting that AOA represented by these two archetypes should be important when AOA abundance is high.

A recent study has provided direct evidence for cooperation of AOA with anaerobic ammonia-oxidizing (anammox) bacteria by provision of nitrite and consumption of oxygen (Yan et al., 2012). Lam et al. (2009) argued that a significant portion of the nitrite for anammox in the ETSP OMZ was produced by ammonia oxidizers, implying community interactions between AOA and anammox bacteria, despite the fact that the known nitrite-producing metabolism of AOA is not possible in the extremely low oxygen conditions of the OMZ. On the other hand, a study on the depth distribution of AOA and anammox bacteria in the Arabian Sea OMZ suggested their niches were vertically segregated (Pitcher et al., 2011), so the chance of interaction between AOA and anammox bacteria there was low. Therefore, it is possible that those archetypes (AOA -9, -12, -15, -23 and -26) that had higher RFR in the ETSP than in the Arabian Sea represented AOA that had interactions with anammox bacteria. Conversely, those archetypes (AOA -4, -10, -17, -19, -21 and -22) that had higher RFR in the Arabian Sea than in the ETSP represented AOA that were independent of anammox bacteria. Understandably, these potential interactions between microbial communities were not reflected by the physicochemical data. However, such interactions *in situ* remain speculative and require experimental verification.

### Conflict of interest statement

The authors declare that the research was conducted in the absence of any commercial or financial relationships that could be construed as a potential conflict of interest.
